# Concurrent Varicella and Herpes Zoster in an Adult Caused by Varicella-Zoster Virus: A Case Report

**DOI:** 10.7759/cureus.99326

**Published:** 2025-12-15

**Authors:** Hanan F Alotaibi, Huda A Alammari, Faten F Altassan

**Affiliations:** 1 Family Medicine, King Abdulaziz Medical City, Jeddah, SAU; 2 College of Medicine, Umm Al-Qura University, Makkah, SAU; 3 College of Medicine, King Saud bin Abdulaziz University for Health Sciences, Jeddah, SAU

**Keywords:** adult, antiviral treatment, co-occurrence, herpes zoster, varicella, varicella-zoster virus

## Abstract

Varicella-zoster virus (VZV), a member of the herpesvirus family, causes two distinct clinical entities: varicella (chickenpox) and herpes zoster (shingles). Varicella predominantly affects children, whereas herpes zoster results from reactivation of latent VZV and is more commonly observed in older adults and immunocompromised individuals. The simultaneous occurrence of varicella and herpes zoster in adults is rare and poses diagnostic and therapeutic challenges. We report the case of a 43-year-old man who presented with a two-day history of fever, a generalized vesicular rash, and an infected wound above the right inguinal region. Physical examination revealed scattered vesicular lesions involving the scalp, forehead, neck, and lower extremities, raising clinical suspicion for concurrent varicella and herpes zoster. Serological testing confirmed acute VZV infection, with elevated IgM and IgG levels. The patient was treated with oral valacyclovir and supportive care, resulting in marked clinical improvement. This case highlights the uncommon co-presentation of varicella and herpes zoster in an adult, which may complicate clinical assessment and management. Although varicella is less common in adults, its coexistence with herpes zoster underscores the importance of early recognition and timely initiation of antiviral therapy. Further studies are warranted to better understand the pathophysiology and optimal management of such dual VZV presentations.

## Introduction

Varicella-zoster virus (VZV), classified as human herpesvirus 3, is responsible for two significant diseases: varicella (commonly known as chickenpox) and herpes zoster (HZ; shingles). Varicella predominantly affects children and is characterized by systemic symptoms such as fever and a generalized vesicular rash. Following primary infection, VZV establishes lifelong latency in the dorsal root ganglia. Reactivation of this latent virus results in HZ, which manifests as a painful, localized vesicular rash typically confined to one or adjacent dermatomes [1,2]. Both HZ and varicella are caused by VZV, and due to its diverse clinical presentations, which are crucial for differential diagnosis, HZ, as a reactivation of latent VZV, has drawn considerable clinical attention. Moreover, complications related to HZ can be severe and potentially fatal.

The likelihood of developing HZ increases with age, particularly in individuals over 50 years, as well as in those who are immunocompromised or under significant physiological or psychological stress [3]. The clinical presentation of HZ can vary widely, and complications such as postherpetic neuralgia can significantly impair quality of life, making early diagnosis and prompt treatment imperative. During the COVID-19 pandemic, VZV reactivation has been a major topic of discussion, particularly following immunization, with several reports describing HZ reactivation as a potential post-vaccination event [2]. Consequently, treatment strategies and vaccination-based prevention remain clinically significant. Different clinical manifestations of HZ may occur, some of which carry a higher risk of adverse outcomes. The literature on HZ continues to evolve, especially in relation to immunocompromised and medically complex individuals.

Although varicella is uncommon in adults, cases of concurrent varicella and HZ have been documented. These instances are particularly noteworthy, as they complicate the clinical picture and pose unique diagnostic and management challenges, potentially increasing the risk of severe complications [4]. Recent advances in understanding the molecular mechanisms of VZV pathogenesis, including the development of novel animal models and genetically engineered recombinants, have provided deeper insights into viral behavior during primary infection and latency [3]. Antiviral therapies such as acyclovir, valacyclovir, and famciclovir have proven effective in the management of both varicella and HZ, while vaccination strategies have been implemented to reduce disease incidence [1]. This case report describes an adult patient presenting with concurrent varicella and HZ, highlighting the need for heightened clinical awareness and timely intervention in managing such complex VZV infections.

## Case presentation

We report the case of a 43-year-old man who presented with a two-day history of fever and a vesicular skin rash, accompanied by a small infected wound above the right inguinal region. On admission, his vital signs were notable for a blood pressure of 105/69 mmHg, heart rate of 96 beats per minute, respiratory rate of 20 breaths per minute, and a temperature of 39.4 °C, later recorded at 38.9 °C, with an oxygen saturation (SpO₂) of 98%.

**Figure 1:**
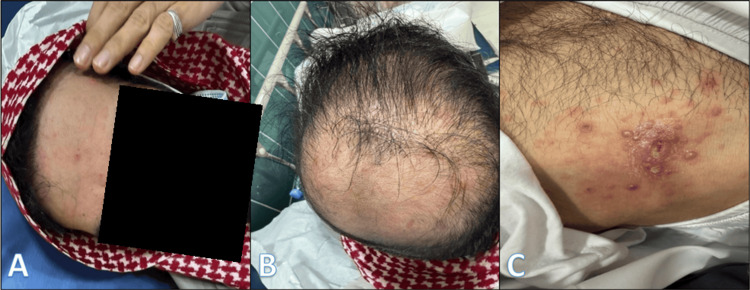
Clinical presentation of vesicular lesions in the patient: (A) forehead, (B) scalp, (C) lateral chest wall.

Physical examination revealed a scattered vesicular rash surrounding the infected wound, as well as involvement of the scalp, forehead, neck, and lower extremities, raising clinical suspicion for concurrent varicella and HZ (Figure 1). The patient reported no ocular symptoms, and an ophthalmology consultation determined that referral was not required at that time, although red flags were identified for ongoing monitoring.

Initial laboratory investigations performed on February 19, 2025, demonstrated a VZV IgG level of 74.22, along with positive herpes simplex virus (HSV) IgG and IgM. Complete blood count results showed a white blood cell count of 6.9 × 10⁹/L, hemoglobin level of 13.6 g/dL, and a reduced lymphocyte count of 0.60 × 10⁹/L. Hepatitis serology was negative for all types, and HIV antigen/antibody testing was negative. Follow-up serological testing revealed a markedly elevated VZV IgG level of 1670 and positive VZV IgM, confirming acute infection. The patient was treated with oral valacyclovir and supportive care, resulting in significant clinical improvement.

## Discussion

VZV infections can range from mild mucocutaneous lesions to potentially fatal neurological complications, including vasculitis and postherpetic neuralgia [5,6]. HZ and varicella represent two distinct clinical manifestations of VZV-induced disease. Varicella typically results from primary VZV infection during childhood, after which the virus establishes latency in the peripheral ganglia. Decades later, reactivation of the latent virus leads to HZ [7]. Nevertheless, cases of varicella in adult populations have been reported in countries such as Japan and India [8-10]. Concurrent varicella and HZ in middle-aged and older individuals has also been documented in several studies [9,10]; however, in many of these reports, the specific pathogen characteristics and genotyping of the causal agent were not identified [4].

Adult varicella outbreaks have not yet been extensively investigated. Although cases of concurrent varicella and HZ remain rare, previous studies have demonstrated that middle-aged and older individuals can present with both conditions simultaneously [9,10]. Similarly, an uncommon case of concurrent varicella and HZ in late adulthood is described in the present report.

In India, 110 young college students were diagnosed with varicella between February 2016 and January 2017 [9]. Additionally, between January 2012 and December 2016, 22 adult foreign nationals from eight different countries contracted varicella in central Tokyo, Japan [10]. A case involving a 54-year-old Japanese woman who developed varicella due to VZV infection was also reported; notably, despite having elevated VZV IgG titers, she experienced secondary VZV infection [11].

This case report highlights the uncommon and complex occurrence of concurrent varicella and HZ in an adult patient, underscoring the importance of including both conditions in the differential diagnosis of vesicular rashes. While varicella is more commonly observed in children, the dual presentation of varicella and HZ in adults poses significant diagnostic and therapeutic challenges, often necessitating a tailored treatment approach. This case further emphasizes the need for increased awareness of such rare manifestations of VZV reactivation, particularly among immunocompromised individuals or those experiencing significant physiological or psychological stress.

## Conclusions

This case highlights the rare occurrence of concurrent varicella and HZ in an adult, which complicates the clinical picture and necessitates careful management. While varicella is less common in adults, its co-occurrence with HZ in this patient underscores the importance of early diagnosis and appropriate antiviral treatment. Further research is needed to explore the clinical implications and optimal management strategies for such dual presentations.
